# A multicenter, open-label, phase 2 study of lenalidomide plus low-dose dexamethasone in Chinese patients with relapsed/refractory multiple myeloma: the MM-021 trial

**DOI:** 10.1186/1756-8722-6-41

**Published:** 2013-06-19

**Authors:** Jian Hou, Xin Du, Jie Jin, Zhen Cai, Fangping Chen, Dao-bin Zhou, Li Yu, Xiaoyan Ke, Xiao Li, Depei Wu, Fanyi Meng, Huisheng Ai, Jingshan Zhang, Honeylet Wortman-Vayn, Nianhang Chen, Jay Mei, Jianmin Wang

**Affiliations:** 1 Department of Hematology, Shanghai Changzheng Hospital, Shanghai 200003, China; 2 Guangdong General Hospital, Guangzhou, China; 3 The 1st Hospital, Zhejiang University, Hangzhou, China; 4 Xiangya Hospital of Central South University, Changsha, China; 5 Peking Union Medical College Hospital, Beijing, China; 6 The 301 Hospital-Chinese PLA General Hospital, Beijing, China; 7 Peking University Third Hospital, Beijing, China; 8 Shanghai 6th Hospital, Shanghai, China; 9 The 1st Affiliated Hospital of Soochow University, Suzhou, China; 10 Nanfang Hospital of Southern Medicine University in Guangzhou, Guangzhou, China; 11 The 307 PLA Hospital, Beijing, China; 12 Celgene Corporation, Summit, NJ, USA; 13 Changhai Hospital, Shanghai, China

**Keywords:** Relapsed/Refractory Multiple Myeloma, Chinese Patients, Lenalidomide, Low-dose Dexamethasone

## Abstract

**Background:**

There is an unmet need for treatment options in Chinese patients with relapsed or refractory multiple myeloma (RRMM). Lenalidomide plus low-dose dexamethasone is effective and generally well tolerated in Caucasian RRMM patients, but no previous study has evaluated this regimen in Chinese RRMM patients.

**Methods:**

MM-021 is a phase 2, multicenter, single-arm open-label registration trial conducted to assess the efficacy, safety, and pharmacokinetics of lenalidomide plus low-dose dexamethasone in Chinese patients with RRMM. Patients with ≥1 prior antimyeloma therapy received lenalidomide plus low-dose dexamethasone until disease progression or discontinuation. Follow-up of surviving patients continued for ≥1 year after enrollment. The lenalidomide dose was 25 mg/day, and was adjusted according to baseline renal function. Most patients had advanced disease (85.6% had Durie*-*Salmon stage III) and were heavily pretreated (56.7% had received ≥4 prior regimens; 69.5% prior thalidomide and 63.1% prior bortezomib); 5.3% had immunoglobulin D (IgD) disease.

**Results:**

The safety population comprised 199 eligible patients. In the efficacy population (n = 187), the disease control rate (at least stable disease) was 94.7%, and the overall response rate (at least partial response) was 47.6%. High response rates were also achieved in patients who had renal impairment and in those with IgD disease. After a median study follow-up of 15.2 months, the median response duration was 8.8 months (range, 0.4–18.8 months) and median progression-free survival was 8.3 months (95% CI 6.5–9.8). The most common grade 3–4 adverse events (AEs) were anemia (26.1%), neutropenia (25.1%), thrombocytopenia (14.6%), pneumonia (13.1%), leukopenia (9.5%), and decreased neutrophil count (8.5%). AEs led to lenalidomide dose reduction and/or interruption in 40.2% of patients, and treatment discontinuation in about 9% of patients. The pharmacokinetic profile of lenalidomide was similar to that reported in Caucasian and Japanese patients.

**Conclusions:**

Lenalidomide plus low-dose dexamethasone was associated with a high response rate and acceptable safety profile in heavily pretreated Chinese patients with RRMM, including those with renal impairment and IgD subtype. These findings highlight the clinical potential of this regimen in Chinese RRMM patients who have exhausted current treatment options.

**Trial registration:**

China State Food and Drug Administration (SFDA) registration (CTA reference numbers: 209 L10808; 209 L10809; 209 L10810; and 209 L10811) and ClinicalTrials.gov identifier: NCT01593410.

## Introduction

In China, the annual incidence of hematological malignancies, including multiple myeloma (MM), is estimated to be approximately 2 per 100,000 people [1-3]. The introduction of innovative therapies, such as proteasome inhibitors and immunomodulatory drugs, has improved the prognosis for MM patients worldwide [4]. The Chinese Multiple Myeloma Working Group treatment guidelines currently recommend bortezomib- and thalidomide-containing regimens for newly diagnosed MM [5], although only bortezomib has been approved for the treatment of MM in China. As MM patients will eventually relapse or become refractory to current treatments, there is a need for new therapeutic agents to offer more options when this occurs.

Lenalidomide in combination with dexamethasone has recently been approved by the Chinese authorities as a treatment for patients with relapsed or refractory MM (RRMM) who have received ≥1 prior therapy. In this setting, lenalidomide plus high-dose dexamethasone has been shown to be superior to high-dose dexamethasone alone in phase 3 studies [6,7]. The combination regimen has been shown to be safe and effective in various RRMM patient populations, including those in North America, Australia, Europe, Israel, and Japan [6-8]. The pharmacokinetic (PK) profile of lenalidomide does not appear to be sensitive to ethnic factors and has been reported as similar in Japanese MM patients, healthy Caucasian volunteers [8], and MM patients [9] over the clinical dose range of 5–25 mg/day. In a study conducted by the Eastern Cooperative Oncology Group (ECOG) in patients with newly diagnosed MM, 1-year survival was superior with lenalidomide plus low-dose dexamethasone compared with lenalidomide plus high-dose dexamethasone [10]. However, the lenalidomide plus low-dose dexamethasone regimen has not been studied in patients with RRMM. The MM-021 China Registration study was conducted to assess the efficacy, safety, and PK profile of lenalidomide plus low-dose dexamethasone in Chinese patients with RRMM. The study is registered at ClinicalTrials.gov (NCT01593410) and with the China State Food and Drug Administration (SFDA) (CTA reference numbers: 209 L10808; 209 L10809; 209 L10810; and 209 L10811).

## Methods

MM-021 was designed and monitored in accordance with the ethical principles of good clinical practice and the Declaration of Helsinki, and was approved by an Independent Ethics Committee. Patients provided written informed consent prior to enrollment. The primary objective was to determine the efficacy of lenalidomide plus low-dose dexamethasone in Chinese patients with RRMM. The secondary objectives were to determine the safety of lenalidomide plus low-dose dexamethasone and the PK of lenalidomide when administered alone or with low-dose dexamethasone in this population. The co-authors analyzed and interpreted data; all authors had access to the primary clinical trial data.

### Study design

MM-021 is phase 2, open-label, multicenter, single-arm trial that enrolled RRMM patients between September 12, 2010 and June 3, 2011. The data cutoff date for the final analysis was September 26, 2012. For the PK assessment, a target sample size of 10 patients was required to obtain reasonable estimates of systemic exposure to lenalidomide in patients with adequate renal function. The first 11 enrolled patients aged ≤75 years who had a baseline creatinine clearance (CL_Cr_) of ≥60 ml/min were included in the PK assessment cohort. Subsequent patients were enrolled in the treatment cohort without PK assessments. Lenalidomide plus low-dose dexamethasone therapy was given in both cohorts until disease progression or treatment discontinuation. All patients (including those who discontinued treatment for any reason) were followed up for survival and subsequent antimyeloma treatments every 4 months (±7 days) for ≥1 year after enrollment.

### Inclusion and exclusion criteria

Eligible patients were aged ≥18 years, had Durie-Salmon MM stage II or III, and had disease progression after ≥2 cycles of antimyeloma treatment or relapsed with progressive disease after therapy. Other inclusion criteria were: measurable levels of M-protein in serum (≥0.5 g/dl) or urine (≥0.2 g/24 hours); an ECOG Performance Status score of ≤2; an absolute neutrophil count (ANC) of ≥1000 cells/mm^3^ (≥1.0 × 10^9^/l); a platelet count of ≥50,000/mm^3^ (≥50 × 10^9^/l); a serum transaminase level of ≤3.0 × the upper limit of normal; and a serum total bilirubin level of ≤2.0 mg/dl (≤34 μmol/l). Females of childbearing potential were eligible if they agreed to use ≥2 forms of reliable contraception, have two medically supervised pregnancy tests, and not breastfeed during the study. Males were also required to use contraception throughout study drug therapy, during any dose interruption, and for ≥28 days following study drug discontinuation.

Patients were excluded if they had non-secretory MM; renal failure requiring dialysis; significant active cardiac disease within the past 6 months (including angina requiring medical intervention, uncontrolled hypertension, myocardial infarction, New York Heart Association class II–IV congestive heart failure, or unstable angina); history of deep-vein thrombosis (DVT) or pulmonary embolism (PE) within the past 12 months; prior malignancy; hypersensitivity to thalidomide or dexamethasone; or prior use of lenalidomide.

Patients at high risk of venous thromboembolism (VTE) received anticoagulation therapy with a low-molecular-weight heparin or full-dose warfarin for at least the first 4 months, followed by low-dose aspirin (70–100 mg/day) or continued anticoagulation at the discretion of the treating physician. All other patients received, at the discretion of the treating physician, either oral low-dose aspirin or another prophylactic antithrombotic treatment during their participation in the study. Patients unable or unwilling to undergo antithrombotic prophylactic treatment were not eligible to participate in this study.

### Treatment

Commercial formulations of lenalidomide and dexamethasone were supplied by Celgene Corporation. Lenalidomide was packaged in 11- and 21-count blister cards for the 5, 10, 15, and 25 mg capsules. Dexamethasone was supplied as 4 mg tablets. Lenalidomide dosing followed the approved product labels [11,12]. The dose of dexamethasone was based on data from a previous randomized study which reported that a lower dose of dexamethasone combined with lenalidomide was effective in the treatment of MM, and was associated with better tolerability compared with lenalidomide and high-dose dexamethasone [10].

Lenalidomide was given orally once daily on days 1–21 of each 28-day cycle; the starting dose was 25 mg/day for patients with normal renal function (CL_Cr_ ≥60 ml/min), 10 mg/day for those with mild-to-moderate renal insufficiency (CL_Cr_ ≥30 to <60 ml/min), and 15 mg every other day for those with severe renal insufficiency (CL_Cr_ <30 ml/min) [13,14]. The dose was modified in patients who experienced grade 4 neutropenia or thrombocytopenia, febrile neutropenia, or any grade ≥3 lenalidomide-related adverse event (AE). When symptoms resolved to grade ≤2, lenalidomide was decreased by one dose level (dose levels decrements of 5 mg) at the start of the next cycle; for neutropenia, granulocyte colony-stimulating factor could be initiated and the dose maintained. Lenalidomide was discontinued for grade 4 rash, grade 4 peripheral neuropathy, or grade ≥3 DVT/PE. Patients aged ≤75 years received oral dexamethasone 40 mg/day on days 1, 8, 15, and 22 of each 28-day cycle. Patients aged >75 years received oral dexamethasone 20 mg/day on days 1, 8, 15, and 22 of each 28-day cycle. Dexamethasone dose levels were 40, 20, 12, 8, and 4 mg; dosing was reduced by one level in patients with grade ≥2 confusion, mood alteration, or muscle weakness; and grade ≥3 dyspepsia, edema, or hyperglycemia. The drug was discontinued in the event of acute pancreatitis. Patients in the PK cohort had PK assessments during the first 8 days of cycle 1 and did not take dexamethasone on Day 1 of cycle 1. Blood samples were collected pre-dose and at 0.5, 1, 1.5, 2, 3, 4, 6, 9, 12, and 24 hours post-dose on Days 1, 7, and 8.

### Outcomes and endpoints

The primary endpoint was best overall response rate (ORR), assessed using European Group for Blood and Marrow Transplantation (EBMT) criteria [15], and responses were also assessed by an Independent Response Adjudication Committee (IRAC). Very good partial response (VGPR) was added as a subcategory of partial response (PR), under the International Myeloma Working Group criteria [16]. Secondary endpoints included progression-free survival (PFS), response duration, safety, and PK parameters of lenalidomide. AEs were coded according to the Medical Dictionary of Regulatory Activities version 14.0, and severity was graded according to the National Cancer Institute Common Terminology Criteria for Adverse Events version 4.0. PFS was calculated as the time between study enrollment and first documented progressive disease or death, whichever occurred first, based on EBMT criteria. Patients who withdrew from the study were censored on the date of their last response assessment. Response duration was calculated as the time from the first response (PR or better) to the first documented progressive disease or death, whichever occurred first. Time to progression (TTP) was defined as time from enrollment to first documented progressive disease. Time to response was defined as the time from enrollment to the first documented response (PR or better). Concentrations of lenalidomide in plasma were determined by a validated liquid chromatography-tandem mass spectrometry method [17].

### Statistical analyses and sample size

Target accrual was 194 patients, including 184 patients in the treatment cohort and 10 patients in the PK cohort. For the PK cohort, the target sample size was 10 patients, to obtain reasonable estimates of systemic exposure to lenalidomide in patients with adequate renal function.

For this analysis, the primary population (n = 187) included the first 176 patients enrolled in the treatment cohort and the 11 patients enrolled in the PK cohort. The efficacy-evaluable population (n = 187) included all patients who took ≥1 dose of the study drug, had measurable disease at baseline, and had ≥1 post-baseline response assessment. To provide the most complete safety information, the safety population (N = 199) included all patients who had enrolled and received ≥1 dose of study drug before data cutoff (September 26, 2012).

The Kaplan–Meier method was used to estimate the survival distribution function for PFS, response duration, and overall survival. The effect of dexamethasone on lenalidomide exposure at steady state was evaluated by comparing area under the plasma concentration–time curve (AUC), AUC from time 0 to infinity (AUC_∞_), and maximum observed plasma concentration (C_max_) between Day 7 (lenalidomide alone) and Day 8 (lenalidomide plus low-dose dexamethasone), using an analysis of variance model (ANOVA) with treatment as a fixed effect and patient as a random effect on the natural log-transformed parameters.

## Results

### Patient disposition

**Figure 1:**
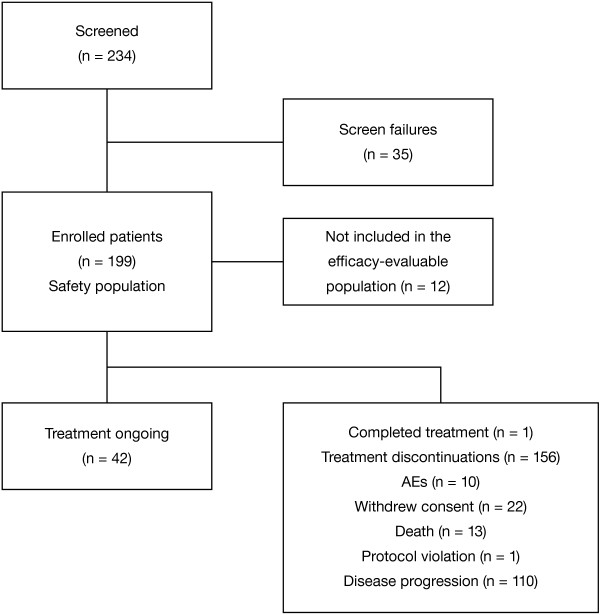
Patient disposition.

**Figure 2:**
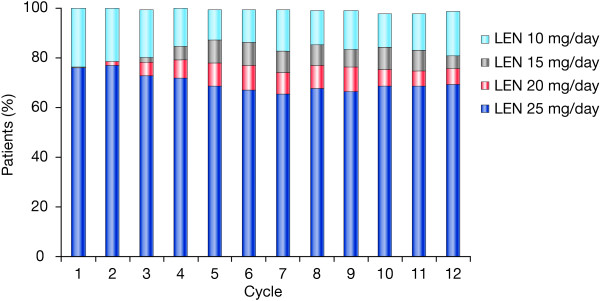
Dose of lenalidomide (LEN) over 12 treatment cycles.

A total of 199 patients were enrolled in the study and were evaluable for safety (Figure 1). At the time of the final analysis, 42 patients continued to receive study treatment, 156 had discontinued, and 1 patient had completed treatment. Treatment discontinuations were due to progressive disease (n = 110), consent withdrawal (n = 22), death (n = 13), AEs (n = 10), or protocol violation (n = 1). The final efficacy analysis was based on the primary efficacy population of 187 patients (176 in the treatment cohort and 11 in the PK cohort). The median treatment duration was 8.3 months (range 0.9–22.9 months) and the median number of cycles was 9 (range 1–25). The median durations of lenalidomide and dexamethasone treatment were 7.8 months (range 0.1–22.9 months) and 7.6 months (range 0.0–22.8 months), respectively. The median relative dose intensity (actual dose intensity/planned dose intensity) was 1.0 for both lenalidomide (range 0.3–2.3) and dexamethasone (range 0.4–7.0), suggesting that most patients received their dose of study medication as planned over the course of the study. The proportion of patients receiving the 25, 20, 15, and 10 mg doses of lenalidomide over 12 progressive treatment cycles is shown in Figure 2. The majority of patients per treatment cycle remained on the 25 mg/day starting dose.

### Baseline demographics

Patient characteristics at baseline are shown in Table 1. The median age of patients in the primary efficacy population was 60.0 years (range 35.0–81.0 years), 71.1% were aged ≤65 years, and 62.0% were male. Most patients in the primary population had advanced disease (85.6% had Durie-Salmon MM stage III) and were heavily pretreated; 56.7% had received ≥4 prior regimens and most had received prior thalidomide (69.5%), bortezomib (63.1%), or both (44.9%). In addition, 5.4% of patients had the immunoglobulin D (IgD) subtype of MM. Most patients (66.8%) had normal renal function (CL_Cr_ ≥60 ml/min), 26.7% had mild-to-moderate impairment (CL_Cr_ ≥30 to <60 ml/min), and 6.4% had severe impairment (CL_Cr_ <30 ml/min). Baseline patient characteristics (including renal function) were comparable between the primary and the safety populations.

**Table 1:** Baseline patient characteristics

	**Primary efficacy population (n = 187)**	**Safety population (N = 199)**
Median age, years (range)	60 (35–81)	59 (35–81)
Age distribution, n (%)		
≤65 years	133 (71.1)	142 (71.4)
>65 years	54 (28.9)	57 (28.6)
Male, n (%)	116 (62.0)	125 (62.8)
Durie-Salmon baseline MM stage, n (%)		
I	9 (4.8)	9 (4.5)
II	18 (9.6)	19 (9.6)
III	160 (85.6)	171 (85.9)
ECOG Performance Status score, n (%)		
0	69 (36.9)	73 (36.7)
1	92 (49.2)	99 (49.7)
2	26 (13.9)	27 (13.6)
Renal function (creatinine clearance), n (%)		
Normal (≥60 ml/min)	125 (66.8)	131 (65.8)
Mild-to-moderate impairment (≥30 to <60 ml/min)	50 (26.7)	54 (27.1)
Severe impairment (<30 ml/min)	12 (6.4)	14 (7.0)
Median number of prior antimyeloma therapies, n (range)	4 (1–15)	4 (1–15)
Number of prior antimyeloma therapies, n (%)		
1–3	81 (43.3)	86 (43.2)
4–6	64 (34.2)	68 (34.2)
7–9	29 (15.5)	32 (16.1)
10–15	13 (7.0)	13 (6.5)
Prior use of thalidomide or bortezomib, n (%)		
Thalidomide	130 (69.5)	137 (68.8)
Bortezomib	118 (63.1)	127 (63.8)
Thalidomide and bortezomib	84 (44.9)	90 (45.2)
Type of MM, n (%)		
IgA	50 (26.7)	55 (27.6)
IgD	10 (5.4)	10 (5.0)
IgG	107 (57.2)	112 (56.3)
IgM	1 (0.5)	1 (0.5)
Missing	19 (10.2)	21 (10.6)

Abbreviations: *ECOG* Eastern Cooperative Oncology Group, *Ig* immunoglobulin, *MM* multiple myeloma.

### Efficacy

The primary efficacy endpoint of best ORR (defined as PR or better by IRAC assessment using EBMT criteria) in the efficacy-evaluable population (n = 187) was 47.6%, including 23 patients (12.3%) with a VGPR and 7 patients (3.7%) who had a complete response (CR) (Table 2). An additional 88 patients (47.1%) achieved stable disease (SD), resulting in a disease control rate (SD or better) of 94.7%. The median time to response was 1.9 months (range 0.9–10.2 months).

**Table 2:** Best response as assessed by the Independent Response Adjudication Committee (IRAC)

	**Efficacy-evaluable population (n = 187)**
Overall response (CR + VGPR + PR), n (%) [95% CI]	89 (47.6) [40.4–54.8]
CR, n (%)	7 (3.7)
PR, n (%)	82 (43.9)
VGPR, n (%)	23 (12.3)
Stable disease, n (%)	88 (47.1)
Progressive disease, n (%)	10 (5.3)
Median duration of response, months (range)	8.8 (0.4–18.8)
Median time to response, months (range)	1.9 (0.9–10.2)

Abbreviations: *CR* Complete response, *PR* Partial response, *VGPR* Very good partial response.

**Figure 3:**
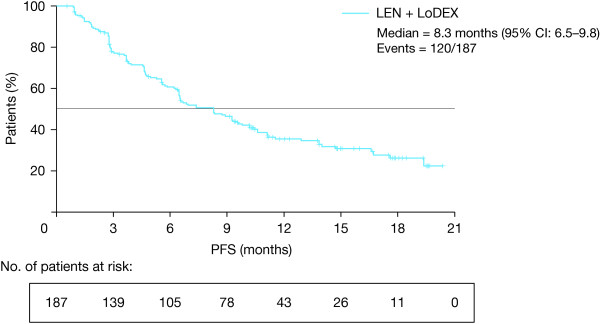
Kaplan–Meier curve of progression-free survival (PFS) for the primary efficacy population (n = 187).

Secondary efficacy endpoints included PFS and response duration in the efficacy-evaluable population (n = 187). With a median PFS follow-up of 6.5 months (range 0.6–20.3 months), the median PFS was 8.3 months (95% CI 6.5–9.8) (Figure 3). The majority of patients (60.6%) were progression-free at 6 months, and approximately half the patients (46.4%) remained progression-free at 9 months. The median duration of response was 8.8 months (range 0.4–18.8 months).

Additional endpoints included response rates in various subgroups based on baseline characteristics. The response rate was 58.0% in patients who had earlier treatment (≤2 prior therapies; n = 50) and 43.8% in those who had received later treatment (>2 prior therapies; n = 137). Regarding the type of prior therapy, the response rate was 46.2% in those who had previously received thalidomide (n = 130), 47.5% in those who had previously received bortezomib (n = 118), 45.2% in those who had received both thalidomide and bortezomib (n = 84), and 47.8% in those who had received neither prior thalidomide nor bortezomib (n = 23). The response rate was comparable across patient subgroups with different degrees of renal impairment. Based on renal function, the ORR was 50.4% in patients with normal renal function (n = 125), 42% for those with mild-to-moderate renal impairment (n = 50), and 41.7% in those with severe renal impairment (n = 12). Patients with IgD MM (n = 10), had a higher response rate than the overall efficacy population (response rate 70.0%, including 10% CR and 50% VGPR). However, the median duration of response was shorter in patients with IgD MM compared with the overall efficacy population, with a median duration of response of 6.6 months (range, 4.6–10.1 months) vs. 8.8 months (range 0.4–18.8 months) in the efficacy-evaluable population.

### Safety

The most common treatment-emergent AEs (all grades) were anemia (60.3%), decreased neutrophil and white blood cell counts (41.2% and 32.7%, respectively), neutropenia (35.7%), thrombocytopenia (21.6%), fatigue (19.6%), upper respiratory tract infection (19.6%), and pneumonia (18.6%). Grade 3–4 AEs were reported in 139 of 199 patients (69.8%). The most common treatment-emergent grade 3–4 AEs were anemia (26.1%), neutropenia (25.1%), thrombocytopenia (14.6%), pneumonia (13.1%), leukopenia (9.5%), and decreased neutrophil count (8.5%) (Table 3). One patient developed grade 3 febrile neutropenia. No grade 3–4 peripheral neuropathy was reported. Serious AEs were reported in 58 patients in the safety population (29.1%). The most common serious AEs were pneumonia (11.6%), followed by thrombocytopenia (3.5%), cardiac failure (2.0%), anemia (2.0%), and renal failure (1.5%).

**Table 3:** Grade 3–4 treatment-emergent adverse events (AEs) reported in ≥2% of patients

	**Safety population (N = 199)**
Hematologic AEs, n (%)	
Anemia	52 (26.1)
Neutropenia	50 (25.1)
Thrombocytopenia	29 (14.6)
Leukopenia	19 (9.5)
Non-hematologic AEs, n (%)	
Pneumonia	26 (13.1)
Upper respiratory tract infection	8 (4.0)
Fatigue	8 (4.0)
Hypokalemia	14 (7.0)
Hyperglycemia	5 (2.5)
Hypocalcemia	5 (2.5)
Investigations	
Neutrophil count decreased	17 (8.5)
Platelet count decreased	14 (7.0)
White blood cell count decreased	14 (7.0)
Discontinuation due to AEs, n (%)	18 (9.0)

AEs of special interest included VTE, tumor lysis syndrome, and second primary malignancy (SPM). Only 1 patient (0.5%) experienced DVT, which was a serious event that led to a dose interruption and lenalidomide dose reduction; the patient subsequently recovered. One other patient (0.5%) experienced tumor lysis syndrome with a fatal outcome. Another patient developed an SPM (solid duodenal tumor) that was not considered to be related to either lenalidomide or dexamethasone.

Treatment-emergent AEs resulted in discontinuation of lenalidomide treatment in 18 patients (9%). Cardiac failure, thrombocytopenia, pneumonia, and constipation led to discontinuation of lenalidomide in 2 patients each. No other treatment-emergent AE resulted in the discontinuation of lenalidomide in >1 patient. AEs resulted in dose reduction or interruption of lenalidomide in 80 patients (40.2%) and of dexamethasone in 87 patients (43.7%). The most common reasons for dose reduction or interruption of lenalidomide or dexamethasone were neutropenia (16.6% and 11.6%, respectively), thrombocytopenia (7.5% and 5.0%), decreased neutrophil count (7.5% and 6.0%), pneumonia (7.0% and 8.5%), decreased platelet count (4.0% each), pyrexia (4.0% each), and fatigue (3.5% and 2.0%). In total, 20 patients (10.1%) in the safety population received blood transfusions and 5 patients (2.5%) were treated for anemia with growth factors.

As of the date of the final analysis, 77 patients in the safety population had died either during treatment or during the follow-up phase of the study. Twenty-five of these patients died within 30 days of the final dose of study medication. The most common cause of death was MM/disease progression (17 patients); multi-organ failure (6 patients), cardiac failure and lung infection (4 patients each in safety population); intracranial hemorrhage, renal failure, and respiratory failure (3 patients each); and cardiopulmonary failure, pneumonia, septic shock, and cerebral hemorrhage (2 patients each).

### Pharmacokinetics

**Figure 4:**
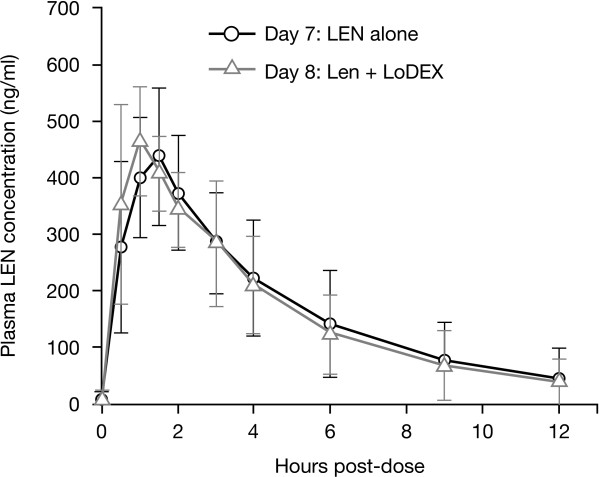
Mean (± standard deviation) plasma lenalidomide (LEN) concentrations in the absence (Day 7) and presence (Day 8) of low-dose dexamethasone (LoDEX).

The first 11 patients enrolled in the study who were aged ≤75 years and had a baseline CL_Cr_ of ≥60 ml/min were enrolled in the PK cohort. When administered to Chinese patients with RRMM under fasting conditions, lenalidomide was absorbed and eliminated rapidly with a median time to peak concentration of approximately 1 hour and median half-life of approximately 3 hours after both single and multiple doses (Table 4). There were no considerable differences in any of the PK parameters between a single dose (Day 1) and multiple doses (Day 7) of lenalidomide administration. The accumulation ratio between Days 1 and 7 was 0.8 and 0.9 for C_max_ and AUC, respectively, demonstrating that lenalidomide did not accumulate in plasma with multiple doses. There were also no apparent differences in any of the multiple-dose PK parameters for lenalidomide alone (Day 7) or lenalidomide plus low-dose dexamethasone (Day 8) (Figure 4). Lenalidomide undergoes limited metabolism and has a primarily renal route of elimination; the lenalidomide PK parameters observed in this study were similar to those previously reported in Caucasian and Japanese patients with RRMM and normal renal function (Table 5) [8,9], Celgene data on file].

**Table 4:** Plasma lenalidomide pharmacokinetic (PK) parameters when administered alone or in combination with dexamethasone (PK cohort, n = 11)

**PK parameter**^**a**^	**Day 1**	**Day 7: lenalidomide alone**	**Day 8: lenalidomide plus low-dose dexamethasone**
	**(n = 11)**	**(n = 10)**	**(n = 10)**
T_max_ (h)	0.93 (0.50–3.17)	1.50 (0.50–3.08)	1.00 (0.50–2.98)
C_max_ (ng/ml)	574 (28.3)	478 (19.3)	494 (19.9)
AUC_t_ (h•ng/ml)	2323 (40.1)	1963 (36.6)	2093 (41.2)
AUC_∞_ (h•ng/ml)	2403 (41.2)	2141 (45.4)	2162 (42.6)
t_1/2_ (h)	3.34 (41.9)	2.79 (32.6)	3.08 (46.8)
CL/F (ml/min)	173 (41.2)	195 (45.4)	193 (42.6)
RAC (C_max_)	NA	0.84 (29.2)	NA
RAC (AUC_τ_)	NA	0.89 (17.9)	NA

^a^Geometric mean (geometric coefficient of variation %) data are presented for all parameters except T_max_ for which median (range) data are presented.

Abbreviations: *AUC* Area under the plasma concentration–time curve, *AUC*_*∞*_ AUC from time 0 to infinity, *CL/F* Apparent total plasma clearance, *C*_*max*_ Maximum observed plasma concentration, *NA* Not applicable, *RAC (AUC*_*τ*_*)* Accumulation ratio between Days 1 and 7 based on AUC, *RAC (C*_*max*_*)* Accumulation ratio between Days 1 and 7 based on C_max_, *t*_*1/2*_ Terminal phase half-life, *T*_*max*_ Time to C_max._

**Table 5:** Comparison of plasma lenalidomide pharmacokinetic parameters (administered with dexamethasone) in different populations of patients with relapsed myeloma and normal renal function (creatinine clearance ≥60 mL/min)

**PK parameter**^**a**^	**Caucasian MM patients**	**Japanese MM patients**	**Chinese MM patients**
	**n = 34**	**n = 12**	**n = 9**
	**(MM-001 and MM-002)**^**c**^	**(MM-017)**^**c**^	**(MM-021)**^**b**^
Median age, years (range)	59 (40–69)	63 (43–66)	55 (44–68)
Median body weight, kg (range)	82 (50–118)	59 (48–75)	65 (54–84)
Median CrCl, ml/min (range)	101 (65–155)	91 (63–135)	95 (63–154)
AUC_∞_ (h•ng/ml)	2124 (28.6)	2305 (23.7)	2202 (30.6)
C_max_ (ng/ml)	487 (35.0)	572 (33.2)	596 (30.2)
T_max_ (h)	1.0 (0.4–4.0)	1.0 (0.4–2.0)	0.93 (0.5–1.0)
CL/F (ml/min)	196 (28.7)	181 (23.7)	184 (30.7)
t_1/2_ (h)	3.18 (20.7)	2.70 (19.3)	3.18 (39.0)
Vz/F (litres)	54.0 (29.5)	41.8 (14.3)	50.7 (28.4)

^a^Geometric mean (geometric coefficient of variation%) data are presented for all parameters except when stated as median (range).

^b^Only includes patients with normal renal function (creatinine clearance ≥60 mL/min).

^c^See references [8,9] and [Celgene data on file].

Abbreviations: *AUC* Area under the plasma concentration–time curve, *AUC*_∞_ AUC from time 0 to infinity, *CL/F* Apparent total plasma clearance, *C*_*max*_ Maximum observed plasma concentration, *CrCL* Creatinine clearance, *t*_*1/2*_ Terminal phase half-life, *T*_*max*_ Time to C_max_, *Vz/F* Apparent volume of distribution during terminal phase after non-intravenous administration.

The addition of dexamethasone had no effect on the plasma exposure to lenalidomide, as indicated by the comparison of AUC and C_max_ on Day 7 (lenalidomide alone) and Day 8 (lenalidomide plus dexamethasone). The 90% CIs for the ratio of geometric means between the two treatments were within the conventionally accepted equivalence limits of 80% and 125% for both AUC and C_max_.

## Discussion

In the previous pivotal, global phase 3 trials (MM-009 and MM-010), the combination of lenalidomide and high-dose dexamethasone demonstrated significantly greater efficacy over dexamethasone alone and was generally well tolerated in treating >700 patients with RRMM [6,7,18]. As the Chinese registration study bridging to the pivotal global trials, the MM-021 phase 2 trial confirmed the efficacy and safety of lenalidomide in combination with lower dose of dexamethasone in Chinese patients with RRMM.

The results of the MM-021 trial are largely consistent with the MM-009 and MM-010 studies, despite having been carried out in a smaller, homogeneous patient population that was more severely ill (>80% had Durie-Salmon MM stage III disease) and more heavily pretreated (91.5% had received ≥2 prior antimyeloma therapies and 57% had received ≥4 prior antimyeloma therapies vs. 62.3% who had received ≥2 prior therapies in MM-009 and MM-010) [19]. The best ORR with lenalidomide plus low-dose dexamethasone in the MM-021 analysis was slightly lower than the best ORR in the MM-009 and MM-010 studies (47.6% vs. 60.6%, respectively) [6,7,18], perhaps explained by dexamethasone dosing and disease severity differences. In the pivotal studies, dexamethasone was given at an intense dose and schedule for the first 4 cycles (40 mg on days 1–4, 9–12, and 17–20; 480 mg per cycle), compared with the low-dose dexamethasone given in the MM-021 study (40 mg on days 1, 8, 15, and 22; 160 mg per cycle). The rationale for using low-dose dexamethasone in the MM-021 trial was based on providing better tolerability while still achieving the synergistic activity of lenalidomide and dexamethasone as shown by Rajkumar et al. [10] in patients with newly diagnosed MM.

Notably, MM-021 patients who had received ≤2 treatments prior to lenalidomide and low-dose dexamethasone had a higher ORR (58%) than the overall efficacy population—closer to the rate reported in the MM-009 and MM-010 studies. These findings suggest that treatment outcomes may be better if lenalidomide and dexamethasone treatment is initiated early (after an initial relapse) rather than after several previous therapies. This observation is consistent with a similar analysis based on the pivotal studies [19].

Importantly, in MM-021 the overall disease control rate (i.e. attainment of SD or better) was 94.7%, which is clinically meaningful considering the high percentage of patients who had a large tumor burden, poor prognosis, and rapidly progressing MM. The analysis for PFS at the data cutoff is limited to a median PFS follow-up of 6.5 months; however, median duration of response in this patient population was 8.8 months, median PFS was 8.3 months, and over half of the patients (60.6%) were progression-free at 6 months. This represents a clinically significant delay in disease progression in a patient population with advanced disease who had relapsed after >2 prior therapies. Moreover, the median PFS in MM-021 is consistent with observations of TTP observed in the MM-009 and MM-010 studies, in which the median TTP was 11.1 and 11.3 months, respectively [6,7].

The MM-021 analysis also included some special patient populations, including those with renal impairment and IgD disease. Lenalidomide undergoes limited metabolism and has a predominantly renal route of excretion; in patients with renal impairment the plasma concentration and half-life of the drug are significantly higher [20]. Therefore, it is important to use lower lenalidomide starting doses in this population. A subanalysis of patients from the MM-009 and MM-010 studies based on renal function status indicated that, even with dose reductions and interruptions, there were no differences in response rates in patients with renal impairment or failure as compared with patients with normal renal function [14]. A prospective study of 50 patients with renal impairment or renal failure has supported these results [13]. In the MM-021 analysis of Chinese MM patients with renal impairment (including 12 patients with severe renal impairment) a high ORR (41.7%) was achieved. These findings, together with previous reports, indicate that with careful monitoring of creatinine levels and AEs, as well as appropriate dose adjustments, lenalidomide plus low-dose dexamethasone is an effective and well-tolerated treatment option for patients with MM who have renal impairment.

The IgD subtype of MM is associated with severe disease, and ORRs in these patients are reportedly lower than rates in patients with other subtypes of MM [21]. Although the IgD subtype typically occurs in <2% of Caucasian MM patients, 5.0% of the patients enrolled in this study had IgD subtype disease. Also, an ORR of 70% was achieved in patients with IgD MM. Although this was a small subgroup (n = 10), the data suggest a trend toward high efficacy of the lenalidomide plus low-dose dexamethasone regimen in patients with IgD MM and a poor prognosis.

The safety data in this study were consistent with the known safety profile of lenalidomide in the RRMM setting. Overall, the main AEs in MM-021 were hematologic and were manageable by dose adjustment of lenalidomide. The most frequently reported grade 3–4 AEs were cytopenias and pneumonia, and consistent with safety findings from the global pivotal phase 3 studies. The incidence of grade 3–4 anemia was higher in this study than in the phase 3 studies, which may be a result of the already high baseline incidence of anemia (87% in MM-021), partly related to underlying disease characteristics. Importantly, lenalidomide was not associated with peripheral neuropathy in this study. As expected, the most common cause of death in MM-021 was MM or complications due to disease progression. The mortality rate and the primary causes of death were consistent with those expected for a population of patients with RRMM. The incidence of grade 3 VTE was lower than previously observed in phase 3 studies (11.4–14.7%) [6,7]. Only 1 patient in this study experienced a VTE event. This may be due to the requirement for all patients to take antithrombotic prophylactic treatment and/or the overall shorter time on study for patients in this trial. Some evidence also suggests that the risk of VTE may be generally lower in Asian populations compared with Caucasian populations [22,23], including among patients with cancer [24].

The PK profile of lenalidomide in Chinese patients was similar to that historically reported in North American MM patients [9]. Lenalidomide was rapidly absorbed and eliminated, with no accumulation in plasma following single or multiple dosing. Furthermore, the mean plasma lenalidomide exposure levels observed in Chinese MM patients who received the 25 mg lenalidomide dose alone were comparable to those historically observed in Caucasian MM patients [20]. Administration of 40 mg of dexamethasone had no effect on the multiple-dose PK profile of lenalidomide, as evidenced by the almost identical plasma concentration–time curves and similar values of the PK parameters for lenalidomide alone and lenalidomide plus low-dose dexamethasone. The PK data are also consistent with previous observations in Japanese [8] and Caucasian patients with RRMM [9], Celgene data on file]. In RRMM patients with normal renal function, lenalidomide is rapidly absorbed and eliminated, with no evidence of accumulation after multiple doses. Furthermore, plasma exposure to lenalidomide (as evidenced by the AUC_∞_,) was similar in the different MM populations [8,9], Celgene data on file]. The consistency of the PK profile with previous studies suggests that the 25 mg/day starting dose of lenalidomide should also be the same in Chinese patients as in other populations. With progressing treatment cycles, the daily dose of lenalidomide was remarkably stable, remaining at 25 mg/day in around 70% of patients; the 20 mg and 15 mg dose levels were infrequently used. Approximately 20% of patients with renal impairment/renal failure started at a dose of 10 mg/day (cycle 1). In addition to the PK results consistent with previous studies, the efficacy results achieved in this Chinese MM patient population largely maintained on stable doses, which supports lenalidomide 25 mg as the appropriate starting dose for patients without renal impairment. The data also support the same dosing guidelines for Chinese patients with renal impairment as for Caucasian patients with renal impairment.

In the US and in western regions of the world, treatment options for patients with RRMM are evolving, although there is no standard recommended regimen. In this setting, the novel agents thalidomide, lenalidomide, and bortezomib are widely used, often in combination with dexamethasone [4,6,7,25-27]. Patients treated with these novel agents have been shown to have significantly improved response rates, PFS, and overall survival compared with a median survival of <2 years at first relapse prior to their introduction [4,6,7,25-27]. New immunomodulatory agents, such as pomalidomide [28,29] and the second-generation proteasome inhibitor carfilzomib [30] are now being introduced in the US and Europe. Until now, lenalidomide was not available for the treatment of patients with RRMM in China, with available treatments limited to standard chemotherapy, and thalidomide- or bortezomib-based regimens [31,32]. The approval of lenalidomide in China therefore expands the treatment armamentarium for Chinese patients with RRMM.

## Conclusions

The findings from this large phase 2 registration bridging study show that lenalidomide plus low-dose dexamethasone led to high ORRs and was generally well tolerated in a Chinese MM patient population, most of whom were heavily pretreated and had advanced RRMM. The ORR was higher in patients who were treated in earlier lines than in more severely ill patients. Patients with renal impairment and the IgD disease subtype also had clinically meaningful response rates. Dosing remained relatively stable over cycles, with AEs generally well-managed by dosing adjustments.

Many patients with advanced RRMM in China have already failed on thalidomide and/or bortezomib treatments and are in need of additional effective regimens to prevent further disease progression. This report suggests that the lenalidomide plus low-dose dexamethasone regimen has the potential to fulfill an unmet medical need for the RRMM population in China.

## Abbreviations

AE: Adverse events; AUC: Area under the plasma concentration–time curve; CLCr: Creatinine clearance; Cmax: Maximum observed plasma concentration; CR: Complete response; DVT: Deep-vein thrombosis; EBMT: European Group for Blood and Marrow Transplantation; ECOG: Eastern Cooperative Oncology Group; Ig: Immunoglobulin; IRAC: Independent Response Adjudication Committee; MM: Multiple myeloma; ORR: Overall response rate; PE: Pulmonary embolism; PFS: Progression-free survival; PK: Pharmacokinetics; PR: Partial response; RRMM: Relapsed or refractory MM; SD: Stable disease; SPM: Second primary malignancy; TTP: Time to progression; VGPR: Very good PR; VTE: Venous thromboembolism.

## Competing interests

Celgene Corporation sponsored this clinical study and was responsible for clinical oversight of the trial. Tigermed was responsible for medical and site monitoring. JH has served as a consultant for and has received honoraria from Novartis, Johnson & Johnson China, MSD, and Celgene; received research funding from Novartis and Johnson & Johnson China; and was a steering committee member of a study sponsored by Novartis. JZ, HWV, NC, and JM are employees and shareholders of Celgene Corporation. The remaining authors declare no competing financial interests.

## Authors’ contributions

JH, ZC, DW, NC, and JM designed the study; JH, XD, ZC, FC, DZ, LY, XK, XL, DW, HA, JM, and JW enrolled patients; JH, ZC, XL, DW, and JM collected data; JH, ZC, DW, JZ, HWV, NC, and JM analyzed and interpreted data; and all authors critically reviewed the manuscript for important intellectual content and approved the final draft.
